# A digital health program for treatment of urinary incontinence: retrospective review of real-world user data

**DOI:** 10.1007/s00192-022-05321-3

**Published:** 2022-08-15

**Authors:** Laura E. Keyser, Jessica L. McKinney, Samantha J. Pulliam, Milena M. Weinstein

**Affiliations:** 1 School of Rehabilitation Sciences, Andrews University, Berrien Springs, MI 49104 USA; 2 Renovia Inc., Boston, MA 02210 USA; 3 Urogynecology and Pelvic Reconstructive Surgery, Tufts Medical Center, Boston, MA 02111 USA; 4 Female Pelvic Medicine and Reconstructive Surgery, Massachusetts General Hospital, Boston, MA 02114 USA

**Keywords:** Urinary incontinence, Digital therapeutics, Real-world evidence

## Abstract

**Introduction and hypothesis:**

To determine the effectiveness of a prescription digital therapeutic (pDTx) in reducing urinary incontinence (UI) symptoms in real-world users.

**Methods:**

This is a retrospective cohort study of real-world data from users of a pDTx designed to guide pelvic floor muscle training(PFMT) between July 1, 2020–December 31, 2021. The primary outcome was UI symptom change as reported via in-app Urogenital Distress Inventory (UDI-6). Included subjects were female, ≥ 18 years with a diagnosis of stress, urgency, or mixed UI who completed the UDI-6 at baseline and 8 weeks. Demographic, symptom, and adherence data were summarized. Paired *t*-test and Wilcoxon signed rank test were used to analyze change in outcomes from baseline to 8 weeks across adherence and UI diagnosis groups.

**Results:**

Of 532 women with UI, 265 (50%) met criteria and were included in the analysis. Mean age was 51.2 ± 11.5 years (range 22–84, *N* = 265). Mean body mass index (BMI) was 27.3 ± 6.2 kg/m^2^ (range 15.2–46.9, *N* = 147). Most participants had stress UI (59%) followed by mixed UI (22%), urgency UI/OAB (11%), and unspecified UI (8%). UDI-6 scores improved by 13.90 ± 15.53 (*p* ≤ 0.001); 62% met or exceeded MCID. Device-reported PFMT adherence was 72% at 4 weeks and 66% at 8 weeks (100% = 14 uses/week). Participants in each diagnosis category reported significant improvement on UDI-6 score from baseline to 8 weeks. No association between UDI-6 score improvement and adherence category, age, BMI, or UI subtype was identified.

**Conclusions:**

This study demonstrates effectiveness of a pDTx in reducing UI symptoms in a real-world setting. Users achieved statistically and clinically significant symptom improvement over an 8-week period.

**Supplementary Information:**

The online version contains supplementary material available at 10.1007/s00192-022-05321-3.

## Introduction

Female urinary incontinence (UI) is a prevalent health condition, experienced by > 60% of women in the US [1]. The most common subtypes are stress, urgency, and mixed UI [2]. Pelvic floor muscle training (PFMT) represents first-line care for all three subtypes of UI [3]. However, many barriers to first-line care have been reported. Women may lack knowledge about proper technique, frequency, and duration of training [4, 5]. A paucity of trained health care providers to adequately supervise and guide first-line care also limits access to treatment [6–8]. In light of these barriers, digital health solutions have been proposed. Findings from several systematic reviews suggest a role for mobile technologies in UI evaluation and treatment, and this is further supported by professional society guidance [9–13]. Emerging data about specific technologies suggest feasibility, patient acceptability, and efficacy [14, 15].

Digital therapeutics (DTx), both prescribed and non-prescribed, represent an emerging area of digital health. DTx utilize software in the improvement of a health function, prevention of a disease, or the treatment or management of a disease or disorder. Prescription digital therapeutics (pDTx) are considered products, devices, internet applications, or other technologies that are indicated for the prevention, management, or treatment of a medical disease, condition, or disorder, require a formal prescription from a qualified clinician, and are regulated (e.g., cleared or approved by the US Food and Drug Administration). Several additional criteria have been described as fundamental to DTx, including patient privacy and security and published, peer-reviewed clinically meaningful outcomes. One additional criterion and new opportunity presented by pDTx is in the generation of real-world evidence (RWE) made possible through the evaluation of real-world data (RWD) that are generated by use of the pDTx outside of clinical trials. RWE can be a complement to the data generated in traditional clinical trials as a mechanism to deliver information on effectiveness [16]. Effectiveness refers to how an intervention performs in the real world, and efficacy represents how well a treatment or intervention achieves its intended purpose under ideal and controlled circumstances, as derived from RCTs [17]. The contrast between efficacy and effectiveness may be particularly useful in therapies involving behavioral interventions, such as PFMT for UI.

A randomized controlled trial concluded that use of a motion-based prescription pDTx is superior to home PFMT alone for the treatment of stress and mixed UI [14]. This study will review RWD collected from this pDTx designed to treat female UI. The primary objective of this study is to determine the effectiveness of the pDTx in reducing urinary symptoms in women with stress, mixed, and urgency UI, including overactive bladder (OAB). Secondary objectives are (1) to evaluate adherence to treatment and its association with symptom improvement and demographic characteristics and (2) to assess outcomes by UI subtype.

## Materials and methods

This study is a retrospective cohort study of RWD from commercial users of a pDTx. All female subjects, age ≥ 18 years with a UI diagnosis who initiated use from July 1, 2020, to December 31, 2021, were included. Those with off-label or non-UI diagnoses were excluded. Users with complete baseline and 8-week Urogenital Distress Inventory Short Form (UDI-6) scores were included in the outcomes assessment. The UDI-6 represents a validated survey tool used to assess presence and bother of UI symptoms, is scored on a 0–100 point scale, and has been validated for electronic collection [18, 19].

The pDTx (The leva Pelvic Health System, Renovia Inc.) consists of an intravaginal motion sensor and an app-based software program that directs exercise performance and dosing, monitors symptoms and outcomes, and provides health education. The motion sensor transmits exercise data to the software program on the user’s smartphone. This pDTx is FDA-cleared for the treatment of stress, mixed, and mild-moderate urgency UI, including OAB and for pelvic floor muscle weakness. The system passively collects usage information and prompts symptom survey completion (UDI-6), enabling remote monitoring of adherence and symptoms. Individuals using the pDTx have access to a care management team to aid in adherence to PFMT or resolve technical issues, and monthly reports are securely delivered to prescribing clinicians to facilitate monitoring of therapeutic adherence and symptomatic change.

Eligible participants were identified by searching the user database for relevant UI diagnosis codes indicating stress UI, urgency UI/OAB, mixed UI, or unspecified UI (e.g., ICD-10 N39.3, N39.41, N32.81, N39.46). App-collected information includes demographics, UDI-6 scores, adherence (e.g., the number of training sessions completed in a specified time frame), and pelvic floor muscle (PFM) performance (duration of contraction, angle measurements). For this review, all user data were de-identified prior to analysis of user characteristics and treatment outcomes. All users completed device registration and provided informed consent for personal and device-related data to be cloud-captured and stored in a HIPAA-compliant manner (Supplement 1). This study was exempted from review by Western Institutional Review Board.

Participant demographics, diagnosis, UDI-6 survey scores, and adherence data were summarized. Paired *t*-tests and Wilcoxon signed rank tests were used to evaluate UDI-6 outcomes. UDI-6 scores were converted to UDI long form scores [18], and the proportion who achieved the minimum clinical important difference (MCID) of 11 points was determined [20]. Adherence was categorized into three groups: < 5 uses/week; 5–9 uses/week; 10 + uses/week; 14 uses/week or twice daily use represents 100% adherence. Age, BMI, and UDI-6 score change were analyzed by adherence category. A linear regression model using the UDI-6 score change outcome variable and UI subtype, adherence, age, and BMI as independent variables was applied to the data. Adverse events and serious adverse events were monitored and recorded. All analyses were performed using SAS^©^ 9.04 and R 4.1.3.

## Results

**Fig. 1:**
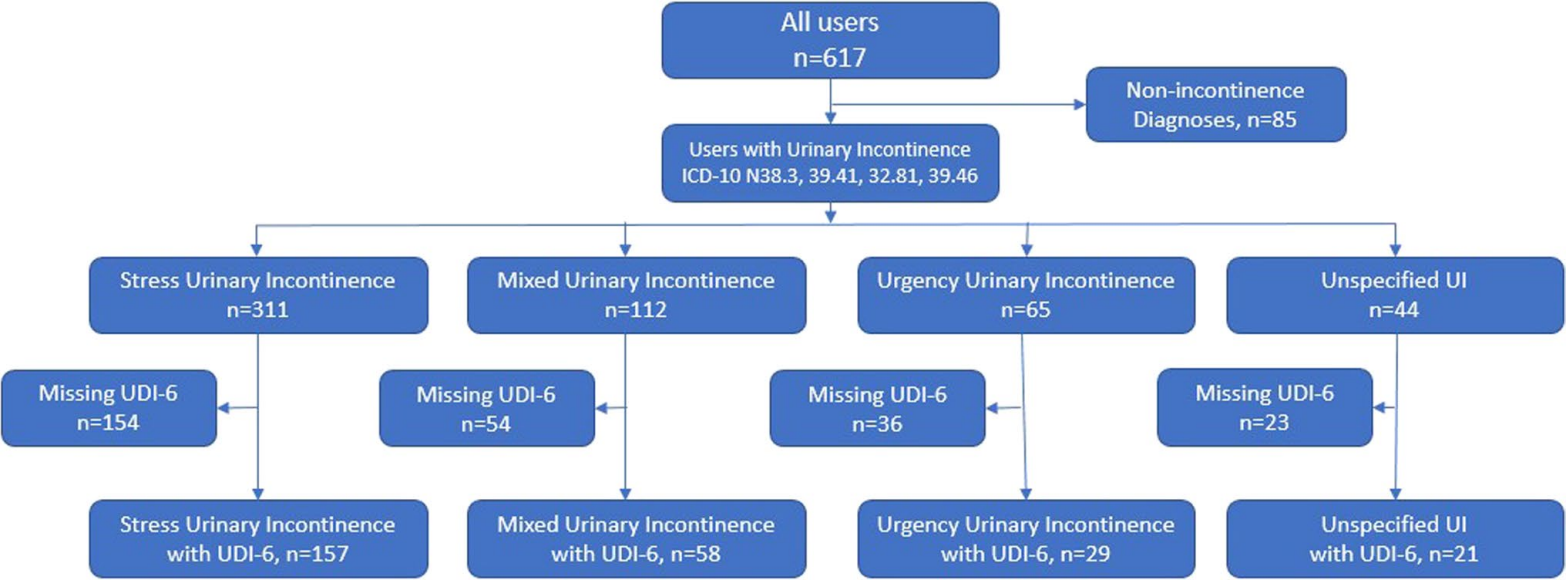
Flow diagram of included participants

Of 617 pDtx users, 532 had diagnoses of UI, and 265 (50%) completed UDI-6 surveys and were included in the analysis (Fig. 1). Table 1 provides baseline demographics and clinical characteristics of this cohort. Mean age was 51.2 ± 11.5 years (range 22–84, *N* = 265). Mean body mass index (BMI) was 27.3 ± 6.2 kg/m^2^ (range 15.2–46.9, *N* = 147). Of those who self-reported race (*N* = 144), 4.9% (13) identified as Black/African American, 1.1% (3) Asian, 44.2% (117) White, and 3.4% (9) Other/Unknown. Most participants had stress UI (59%) followed by mixed UI (22%), urgency UI/OAB (11%), and unspecified UI (8%).

**Table 1:** Summary of demographic and clinical characteristics

	N	Mean	SD
Age (years)	264	51.17	11.49
BMI (kg/m²)	147	27.27	6.17
	N	Frequency	Percent
Race	265		
Black/African American		13	5%
Asian		3	1%
White		117	44%
Other/unknown		9	3%
Missing		123	46%
UI diagnosis	265		
Stress UI		157	59%
Mixed UI		58	22%
Urgency UI/OAB		29	11%
Unspecified		21	8%
Adherence	265		
< 5 uses/week		25	9%
5–9 uses/week		105	40%
10 + uses/week		135	51%

*Abbreviations*: *BMI *body mass index, *UI* urinary incontinence

Overall, UDI-6 scores improved by 13.90 ± 15.53 points from a baseline mean of 43.77 ± 18.99 points to an 8-week mean of 29.87 ± 18.30 points (*p* ≤ 0.0001). At 4 weeks, mean UDI-6 scores were 33.29 ± 19.79 (*p* < 0.001, *n* = 255). Among the 265 participants, 78% (208) reported symptom improvement by week 8; 11% (29) reported no change. Of 11% (28) who indicated worse symptoms, mean UDI-6 score change was 12.20 ± 8.01 points. After conversion of UDI-6 scores to the UDI long form, mean score change was 26.41 ± 29.51 points; 62% met or exceeded MCID of 11 points. Device-reported cumulative PFMT adherence was 72% at 4 weeks and 66% at 8 weeks (100% = 14 uses/week).

Adherence was categorized according to low (< 5 uses/week), medium (5–9 uses/week), and high (10 + uses/week). Table 2 reports age, BMI, and UDI-6 score change by adherence category. Users in the high adherence category were significantly older than users in the other categories (*p* = 0.0002). The low adherence group had a higher BMI compared to medium and high adherence groups, though this was not statistically significant (*p* = 0.1553). A significant UDI-6 score improvement was seen in all three adherence categories. The greatest UDI-6 score change was seen in the high adherence group (-15.23 ± 15.79 points), though this was not statistically different from the medium and low adherence groups. When UDI long form scores were examined by adherence category, the high and medium adherence groups met the MCID of 11 points (*p* < 0.0001 and *p* < 0.0002, respectively), but the low adherence group did not (*p* = 0.2075). No association between UDI-6 score improvement and adherence category, age, BMI, or UI subtype was identified.

**Table 2:** Patient characteristics by adherence category

		Low adherence < 5 uses/week	Moderate adherence 5–9 uses/week	High adherence 10 + uses/week
Age (years)	N*	25	104	135
	Mean (SD)	48.68 (11.85)	48.13 (9.85)	53.96 (11.97)
	Range	27—75	26—79	22—84
BMI (kg/m²)	N*	13	55	79
	Mean (SD)	26.17 (4.32)	28.53 (6.10)	26.57 (6.39)
	Range	20.55—34.21	18.79—43.42	15.21—46.92
8-Week cumulative adherence	N	25	105	135
	Mean (SD)	22% (8)	54% (11)	84% (8)
	Range	0—33%	37—70%	71—100%
Baseline UDI-6 score	N	25	105	135
	Mean (SD)	46.44 (19.70)	42.86 (19.36)	43.99 (18.65)
	Range	5.56—94.44	0.00—100.00	0.00—100.00
8-Week UDI-6 score	N	25	105	135
	Mean (SD)	35.22 (19.29)	30.03 (18.38)	28.77 (18.02)
	Range	0.00—72.22	0.00—94.44	0.00—94.44
UDI-6 score change	N	25	105	135
	Mean (SD)	-11.22 (14.04)	-12.83 (15.51)	-15.23 (15.79)
	Range	-38.89—11.11	-72.22—38.89	-55.56—84.00
	*p*-value	0.0003	< 0.0001	< 0.0001

^*^Age not reported by 1 participant; BMI not reported by 118 participants

*Abbreviations*: *BMI* body mass index, *UDI-6* Urogenital Distress Inventory Short Form

Table 3 demonstrates responses to individual UDI-6 questions and total scores by UI diagnosis and for the total cohort. Participants in each UI diagnosis category reported significant improvement on total UDI-6 score from baseline to 8 weeks. Absolute mean change score for the stress UI diagnosis was 14.14 ± 15.89 (*p* < 0.0001), for mixed UI was 13.03 ± 13.87 (*p* < 0.0001), and for urgency UI/OAB was 16.28 ± 19.18 (*p* < 0.0001). Participants reported the greatest degree of improvement on urinary frequency, urgency UI, and stress UI leakage symptoms (questions 1–3), and these improvements were significant across UI diagnosis categories.

**Table 3:** Responses to individual UDI-6 questions by diagnosis, mean scores (SD)

		Stress UI (*n* = 157)		Mixed UI (*n* = 58)		Urgency UI/OAB (*N *= 29)		Unspecified UI (*N* = 21)		All UI (*N* = 265)	
UDI-6 Questions		Baseline	8 Week	Baseline	8 Week	Baseline	8 Week	Baseline	8 Week	Baseline	8 Week
Q1	Do you usually experience frequent urination?	1.58 (1.00)	1.04 (0.84)	1.83 (0.98)	1.33 (0.89)	2.12 (0.80)	1.66 (0.72)	1.69 (1.03)	1.19 (1.03)	1.70 (0.99)	1.18 (0.87)
Q2	Do you usually experience urine leakage associated with a feeling of urgency?	1.25 (0.95)	0.87 (0.85)	1.66 (1.07)	1.17 (0.88)	1.88 (1.00)	1.07 (0.70)	1.38 (1.12)	1.06 (1.00)	1.42 (1.01)	0.98 (0.86)
Q3	Do you usually experience urine leakage related to coughing, sneezing, or laughing?	2.10 (0.88)	1.39 (0.86)	1.91 (1.01)	1.36 (0.99)	1.07 (1.22)	0.69 (0.93)	1.57 (0.93)	1.02 (0.81)	1.90 (1.01)	1.28 (0.92)
Q4	Do you usually experience small amounts of urine leakage (drops)?	1.59 (0.91)	1.01 (0.76)	1.65 (0.88)	1.17 (0.84)	1.52 (1.02)	1.00 (0.80)	1.43 (0.98)	1.02 (1.05)	1.58 (0.92)	1.05 (0.80)
Q5	Do you usually experience difficulty emptying your bladder?	0.74 (0.93)	0.51 (0.76)	0.74 (0.97)	0.57 (0.77)	1.24 (1.27)	0.79 (0.82)	0.95 (1.12)	0.81 (1.08)	0.81 (1.00)	0.58 (0.80)
Q6	Do you usually experience pain or discomfort in the lower abdomen or genital region?	0.39 (0.67)	0.28 (0.59)	0.39 (0.68)	0.22 (0.59)	0.86 (1.19)	0.55 (0.87)	0.74 (0.94)	0.62 (0.86)	0.47 (0.78)	0.33 (0.66)
Total UDI-6 score		42.43 (18.20)	28.29 (17.86)	45.40 (18.68)	32.38 (17.42)	48.28 (21.21)	31.99 (16.44)	43.12 (22.35)	31.88 (25.36)	43.77 (18.99)	29.87 (18.30)
	p-value	** < 0.0001**		** < 0.0001**		** < 0.0001**		**0.0005**		** < 0.0001**	

All *p* values listed represent significant findings

*Abbreviations*: *SD*  standard deviation, *UDI-6* Urogenital Distress Inventory, *UI* urinary incontinence, *OAB* overactive bladder

Adverse events were reported by 4.5% of women (28/617), and these included pelvic cramping (1.4%), low back pain (0.5%), UTI (0.5%), and skin irritation (0.5%). There were no severe adverse events.

## Discussion

This study provides RWE to support the safety and effectiveness of this pDTx to treat stress, mixed, and urgency UI/OAB with the majority of participants meeting or exceeding the MCID of 11 points on the UDI-6 outcome measure. These findings compliment recently published data from a randomized controlled trial of the pDTx, which demonstrated superior results when compared to PFMT at home without a device in a population with stress and mixed UI [14]. Together, these results support both the efficacy and effectiveness of this intervention as an at-home treatment option for women with UI and may inform clinical decision-making.

Participants with stress, urgency, and mixed UI saw significant symptom improvement during the study period. Urinary frequency and both stress- and urgency-related leakage symptoms were present in all three UI subtype groups, and these symptoms improved significantly during the 8-week study period across all UI subtypes. PFMT is widely established as a treatment for stress UI but may less often be recommended for urgency and mixed UI subtypes despite professional guidance [3, 21, 22].

We did not identify a statistically significant relationship between adherence and change in urinary symptom severity. While it is intuitively reasonable to expect that consistent PFMT would result in more improved symptoms, it is not clear that the relationship between PFMT and adherence is strictly linear. Cumulative adherence over the study period was high and comparable to reported rates of adherence to home exercise programs [23, 24]. However, our inclusion criteria for outcomes evaluation selected those with baseline and 8-week data, meaning participants in this cohort were more likely to be using the device with enough regularity to complete the UDI-6 survey. Among users in the low adherence group (*n* = 25), it is possible that even less frequent use provided adequate neuromuscular re-education to positively affect functional PFM performance—enough for some users to see symptom improvement.

Strengths of this study include the large cohort, as well as the capability of the pDTx to reliably capture adherence and symptom data in a real-world setting. Study limitations include the challenges of real-world survey data. Among participants with UI, our in-app survey response rate of 50% was greater than other real-world, incontinence-related published response rates ranging from 11 to 34% [25, 26]. Completion of the in-app UDI-6 survey is not required to use the pDTx, and users who complete the survey may differ from those who do not. Thus, selection bias may limit interpretation and generalizability of results. In addition, lower response rates to clinico-demographics such as parity, mode of delivery, and past or current UI interventions limited our analysis of these parameters and their relationship to symptom improvement. It is possible that concurrent UI treatments, such as medications (e.g., anticholinergics, hormonal therapies) or pessaries also influenced outcomes. As with most apps, continuous improvement and updates may result in improved data reporting, which is largely dependent upon patient motivation and engagement, as compared to the financial compensation or direct oversight common in randomized trials. Expansion of the onboarding, educational, and motivational content could further enhance patient engagement and provide more complete data sets moving forward.

This study demonstrates effectiveness of a pDTx in reducing UI symptoms among this cohort of users in a real-world setting. Users with stress, mixed, and urgency UI achieved statistically and clinically significant symptom improvement over an 8-week period. Enhanced data collection of relevant demographic and clinical information will further add to the value and applicability of the data. These results may inform additional research and development, including efforts to improve in-app data collection and promote adherence. Given the opportunity of pDTx, additional work designed to present larger patient cohorts is planned, including application of machine learning to expanded data sets. Clinically validated pDTx designed to treat urinary incontinence in women may help to scale treatment and management of this significant, yet undertreated health condition.

## Supplementary Information

Below is the link to the electronic supplementary material.

Supplementary file1 (PDF 183 KB)

Supplementary file2 (DOCX 33 KB)
