# Correction to: A digital health program for treatment of urinary incontinence: retrospective review of real‑world user data

**DOI:** 10.1007/s00192-023-05596-0

**Published:** 2023-06-27

**Authors:** Laura E. Keyser, Jessica L. McKinney, Samantha J. Pulliam, Milena M. Weinstein

**Affiliations:** 1 School of Rehabilitation Sciences, Andrews University, Berrien Springs, MI 49104 USA; 2 Renovia Inc., Boston, MA 02210 USA; 3 Urogynecology and Pelvic Reconstructive Surgery, Tufts Medical Center, Boston, MA 02111 USA; 4 Female Pelvic Medicine and Reconstructive Surgery, Massachusetts General Hospital, Boston, MA 02114 USA

**Correction to: International Urogynecology Journal (2023) 34:1083–1089** 10.1007/s00192-022-05321-3

The article “A digital health program for treatment of urinary incontinence: retrospective review of real‑world user data”, written by Laura E. Keyser, Jessica L. McKinney, Samantha J. Pulliam, and Milena M. Weinstein, was originally published Online First without Open Access. After publication in volume 34, issue 5, pages 1083–1089 the author decided to opt for Open Choice and to make the article an Open Access publication. Therefore, the copyright of the article has been changed to © The Author(s) 2022 and the article is forthwith distributed under the terms of the Creative Commons Attribution 4.0 International License, which permits use, sharing, adaptation, distribution and reproduction in any medium or format, as long as you give appropriate credit to the original author(s) and the source, provide a link to the Creative Commons licence, and indicate if changes were made. The images or other third party material in this article are included in the article's Creative Commons licence, unless indicated otherwise in a credit line to the material. If material is not included in the article's Creative Commons licence and your intended use is not permitted by statutory regulation or exceeds the permitted use, you will need to obtain permission directly from the copyright holder. To view a copy of this licence, visit http://creativecommons.org/licenses/by/4.0/.

The original article has been corrected.

