# Supplementary material for: A digital health program for treatment of urinary incontinence: retrospective review of real-world user data
Source: Int Urogynecol J. 2022 Aug 15;34(5):1083–9. doi: 10.1007/s00192-022-05321-3 (PMC10284973; doi:10.1007/s00192-022-05321-3)
Supplement: Supplementary file 1 — Supplementary file1 (PDF 183 KB) [file 192_2022_5321_MOESM1_ESM.pdf]

Renovia Inc.
Terms of Use & Privacy Policy

GENERAL TERMS OF USE

The following Terms of Use & Privacy Policy ("**Agreement**") are between Renovia Inc. (referred to as "**Renovia**", "**us**" or "**we**") and you and apply to your use of (including any access to) the www.renoviainc.com, www.knowleva.com, and other Renovia websites (the "**Websites**"), the leva and your leva mobile applications or other mobile applications we make accessible or available for download (the "**Applications**"), or any other web-based communication channels or services created, used, offered or operated by Renovia (collectively the "**Services**"). By using or accessing any of the Services or by otherwise accepting this Agreement, you signify your agreement to all of its terms. If you do not agree to the terms of this Agreement you must not use the Services. We reserve the right to change the terms of this Agreement, in which event we will post the changes on this page. Your use of the Services after such changes are posted will mean that you accept such changes.

1. Ownership; Use

All text, graphics, images, logos, photographs, videos, sounds, music, voiceovers, source and object code, algorithms, software, data, messages, posts, information and other materials appearing on or in the Services ("**Content**") are either owned by us, or provided through an arrangement with third parties. Renovia and any such third parties retain all intellectual and proprietary rights to the Content, and the Content is protected by United States and international intellectual property laws, including, but not limited to copyright. You may view, access, download, use and print (if applicable) the Content for your personal, non-commercial use, provided you retain all copyright and other intellectual property and proprietary notices contained in the original Content on any copy. The Content may not be used in any manner not expressly authorized by Renovia under this Agreement and the limited rights granted to you are revocable at any time, with or without notice to you.

In no event may you sell, transfer, assign, license, sublicense, or modify the Content or display, reproduce, perform, broadcast, modify, adapt, translate, make a derivative version of, sell, distribute, or otherwise use the Content in any way for any public or commercial purpose or provide it to any public or commercial website. You may not decompile, reverse engineer, disassemble, decrypt, attempt to derive the source code or object code underlying the Services, or use any automatic tools to use or access the Services, or to otherwise use or access the Services for any unlawful purpose. You may not use the Services in any way that could overburden, damage or disable the Services and/or impair or interfere with other's use of the Services. Deep linking, framing, and the use or posting of the Content on any other website, web-based communication channel, or in a network or similar environment permitting use or access by multiple users at the same time or for any purpose are expressly prohibited. We reserve all rights with respect to the Services not expressly granted herein and may discontinue the Services at any time, with or without notice to you.

Unless otherwise specified, the Services are intended for adult use in the United States of America. No information should be submitted to or posted to any of the Services by users under 18 years of age without the consent of their parent or legal guardian.

Any unsolicited information you provide to Renovia through your use of the Services or through any other means shall be and remain the exclusive property of Renovia. This includes, but is not limited to, information posted to any public areas of the Services, any ideas for new products or modifications to existing products, and other unsolicited submissions (collectively, "**Unsolicited Information**"). All such Unsolicited Information shall be deemed to be non-confidential and Renovia shall be free to reproduce, use, disclose, and distribute such Unsolicited Information to others without limitation or attribution.

Trademarks, service marks, and logos, including but not limited to leva, Lift, and the stylized name of Renovia ("**Marks**") used and displayed on or in any of the Services are our registered and/or unregistered Marks in the United States of America and elsewhere, and nothing on or in the Services or in this Agreement should be construed as granting any license or right to use any Marks without our express prior written permission specifically for each use. Other product and company names mentioned herein may be service marks, trademarks, or registered service marks or registered trademarks of their respective owners.

2. Third-Party Websites

Renovia may offer certain Services through third-party websites or provide links to third-party websites for your convenience. These third-party websites are not under our control, and we are not responsible for the conduct or availability of or content provided by third-party websites. Use of third-party websites is at your own risk. Before disclosing any personally identifiable information on any website, we encourage you to examine the terms and conditions and privacy policy of each of those websites. Links to third-party websites do not constitute or imply an endorsement by Renovia.

3. Geographic Restrictions

The Services are based in the Commonwealth of Massachusetts and/or other locations in the United States of America ("**USA**") and intended for use, access and/or download only by persons located in the USA. You acknowledge that you may not be able to use, access and/or download all or some of the Services outside of the USA and that use, access and download thereto may not be legal by certain persons or in certain countries. If you use, access and/or download the Services from outside the USA, you are responsible for compliance with local laws.

4. Updates

We may, from time-to-time and in our sole discretion, develop and provide updates to the Services, which may include, but are not limited to, upgrades, bug fixes, patches, other error corrections, and/or the removal or introduction of features, functionality and content (collectively, including related documentation, "**Updates**"). You acknowledge and agree that we have no obligation to provide any Updates or to continue to provide or enable any particular feature, functionality and content.

We may make Updates to the Services automatically or prompt you to make them manually, depending both on the applicable Service and the means by which you use, access or download it. For Services you use, access or download from your mobile device(s), this may depend, in part, on the settings you have selected for your mobile device(s). When your internet connection permits: (a) the Services may automatically download and install all available Updates; or (b) you may receive notice of or be prompted to download and install available Updates.

You shall promptly download and install all Updates and acknowledge and agree that the Services or portions thereof may not properly operate should you fail to do so. You further agree that all Updates will be deemed part of the Services and be subject to all terms and conditions of this Agreement.

5. Reservation of Rights

You acknowledge and agree that the Services are provided under license, and not sold, to you. You do not acquire any ownership interest in the Services under this Agreement, or any other rights thereto other than to use the Services in accordance with the limited license granted, and subject to all terms and conditions of this Agreement. We reserve and shall retain our entire right, title, and interest in and to the Services, including all copyrights, trademarks, and other intellectual property rights therein or relating thereto, except as expressly granted to you in this Agreement.

6. Privacy Policy

For more information concerning what information we collect and what we do with such information, see our Privacy Policy below (which, together with these General Terms of Use, constitute the integral parts of the Agreement). We may obtain and release any information if we believe, correctly or not, that such release is necessary to comply with law, to enforce this Agreement, the Privacy Policy, or to safeguard our property or rights.

7. CONTENT IS INFORMATIONAL ONLY; CONSULT YOUR HEALTH CARE PROVIDERS

YOU UNDERSTAND THAT HEALTH CARE AND TREATMENT ARE COMPLEX SUBJECTS REQUIRING THE SERVICES OF QUALIFIED HEALTH CARE PROVIDERS. THE CONTENT PROVIDED ON OR IN THE SERVICES IS INFORMATIONAL ONLY AND NOT INTENDED AS MEDICAL OR HEALTH CARE ADVICE OR RECOMMENDATIONS TO BE USED FOR DIAGNOSIS, TREAMENT OR FOR ANY OTHER INDIVIDUAL NEEDS. THE CONTENT IS NOT A SUBSTITUTE FOR MEDICAL OR HEALTH CARE ADVICE, RECOMMENDATIONS AND/OR SERVICES FROM A QUALIFIED HEALTH CARE PROVIDER. THE CONTENT MAY NOT BE RELIED UPON IN ANY WAY IN CONNECTION WITH YOUR PERSONAL HEALTH CARE, RELATED DECISIONS AND TREATMENT. ALL SUCH DECISIONS AND TREATMENT SHOULD BE DISCUSSED WITH A QUALIFIED HEALTH CARE PROVIDER WHO IS FAMILIAR WITH YOUR INDIVIDUAL NEEDS.

8. Disclaimer of Warranties and Limitations on Liability

Use of the Services is at your sole risk and Renovia assumes no responsibility for your use or access of the Services or any consequences thereof. Except for any express warranty stated on or in the Services, the Services, its Content, and all items offered for sale are provided on an "AS IS" and "AS AVAILABLE" basis without warranties of any kind. RENOVIA DOES NOT MAKE, AND HEREBY DISCLAIMS, ANY REPRESENTATION, AGREEMENT OR WARRANTY WHATSOEVER, EXPRESS OR IMPLIED, INCLUDING BUT NOT LIMITED TO ANY WARRANTIES OF MERCHANTABILITY, FITNESS FOR A PARTICULAR PURPOSE, TITLE, NONINFRINGEMENT OF THIRD PARTIES' RIGHTS OR ANY WARRANTY THAT THE SERVICES OR CONTENT WILL (i) MEET YOUR REQUIREMENTS; (ii) BE UNINTERRUPTED, TIMELY, SECURE, OR ERROR-FREE; (iii) PROVIDE RESULTS THAT WILL BE EFFECTIVE, ACCURATE, OR RELIABLE; (iv) BE FREE FROM ANY ERRORS OR ANY DEFECTS, OR THAT SUCH ERRORS OR DEFECTS WILL BE CORRECTED.

For information on the Limited Warranty available with respect to products manufactured by Renovia, please visit Renovia’s website (www.renoviainc.com) or contact Renovia at Contact@RenoviaInc.com.

IN NO EVENT SHALL RENOVIA BE RESPONSIBLE FOR ANY DAMAGES OF ANY KIND, INCLUDING WITHOUT LIMITATION ANY DIRECT, INDIRECT, SPECIAL, INCIDENTAL, PUNITIVE OR CONSEQUENTIAL DAMAGES TO ANY PERSON, EVEN IF WE KNOW OR HAVE BEEN ADVISED OF THE POSSIBILITY OF SUCH DAMAGES.

Some jurisdictions do not allow the exclusion of implied warranties or limitation of incidental or consequential damages, so not all of this Section may apply to you. RENOVIA'S LIABILITY IN SUCH JURISDICTIONS SHALL BE LIMITED TO THE MAXIMUM EXTENT PERMITTED BY LAW.

9. Termination of This Agreement

By Us. We reserve the right, in our sole discretion, to suspend or terminate this Agreement and your access to all or any part of the Services or their Content, at any time and for any reason with or without prior notice or liability. We reserve the right to change, suspend, or discontinue all or any part of the Services or their Content at any time with or without prior notice or liability.

By You. Your sole means for terminating this Agreement is to discontinue use of the Services.

Survival. If this Agreement is terminated, all sections shall survive the termination.

10. Miscellaneous

This Agreement (including the incorporated Privacy Policy below) constitutes the entire agreement between you and us, and supersedes all prior agreements or communications. If any provision of this Agreement is found to be invalid by any court, the invalidity of such provision shall not affect the validity of the remaining provisions of this Agreement. No waiver shall be effective against us unless we make it in writing, and no such waiver shall be construed as a waiver in any other or subsequent instance. Headings are for convenience only and have no legal import. This Agreement will be governed by the laws of the Commonwealth of Massachusetts applicable to contracts entered into and performed exclusively in Massachusetts. Any court of competent jurisdiction sitting within Suffolk County, Massachusetts will have exclusive jurisdiction and venue for any dispute arising out of or relating to the Services or this Agreement, and you hereby waive any argument that any such court does not have jurisdiction over you or such dispute or that venue is not appropriate or convenient.

PRIVACY POLICY

This Privacy Policy ("**Privacy Policy**") applies to all information or combinations of information that could reasonably allow you to be identified, collectively, ("**Personal Data**") that is collected through your use of (including any access to) the www.renoviainc.com, www.knowleva.com, and other Renovia websites (the "**Websites**"), the *leva* and *your leva* mobile applications or other mobile applications we make accessible or available for download (the "Applications"), or any other web-based communication channels or services created, used, offered or operated by Renovia (collectively the "**Services**"). Personal Data includes information we can use to specifically identify you such as your name, address, date of birth, telephone number, email address, username, Internet Protocol (IP) address or device identifiers when such address is associated with a specific user.

We encourage you to read this Privacy Policy carefully before using the Services. By installing, using, registering, participating or otherwise accessing the Services, you consent to our practices concerning the collection, processing, use and disclosure of your Personal Data in accordance with this Privacy Policy.

Revisions and Updates

Our commitment to be the leading provider of pelvic digital therapeutic products means that our business will continue to evolve as we introduce new services and features. Because of this, from time to time, our policies will be reviewed and may be revised. Renovia reserves the right to change this Privacy Policy at any time and notify you by posting an updated version of the Privacy Policy on the Websites. It is your responsibility to periodically check the Websites so that you are aware of what information we collect, how we process and use it, and under what circumstances we might disclose it. The amended Privacy Policy will apply between us whether or not we have given you specific notice of any change.

Your Personal Information is Important to Us

Renovia respects the importance of your privacy. You have a right to make your own choices about how your Personal Data is used. We know that when you provide Personal Data to us you are showing your trust, and we take that seriously. Unless you otherwise provide your consent, Renovia will only use your Personal Data as we explain in this Privacy Policy.

Collecting Information About You

We collect Personal Data from you when you voluntarily choose to provide such information such as when you enter your Personal Data on our Websites or when you download and register or otherwise access or use an Application. If you contact us, we may also keep a record of that contact.

By voluntarily providing us with Personal Data, you are consenting to our use of it in accordance with this Privacy Policy. If you provide Personal Data through the use of the Services, you acknowledge and agree that such Personal Data may be transferred from your current location to the offices and servers of Renovia and the authorized third parties referred to herein for the purposes described in this Privacy Policy.

Non-Identifiable Data: When you interact with Renovia through the use of the Services, we may receive and store certain personally non-identifiable information. We may store such information ourselves, or it may be included in databases owned and maintained by affiliates, agents or service providers of Renovia. We may use such information and pool it with other information to track, for example, the total number of visitors to our Websites, the number of visitors to each page of our Websites, the domain names of our visitors' Internet Service Providers (ISPs), technical mobile device data such as device model and name, device operating system and version. It is important to note that no Personal Data is available or used in this process.

In operating the Services, we may use various technologies, including "cookies" and other tools. A cookie is a piece of information given to your web browser or device when you access the Services. Our cookies help provide additional functionality to the Services and help us analyze usage of the Services more accurately. For instance, our Websites may set a cookie on your browser that keeps you from needing to remember and then enter a password more than once during a visit to the Websites. On most web browsers, you will find a "help" section on the toolbar. You can refer to this section for information on how to receive notification when you are receiving a new cookie and how to turn cookies off. We recommend that you leave cookies turned on because they allow you to take advantage of some of the Services' features.

Using and Disclosing Your Personal Information

Our purpose in collecting Personal Data about you is to:

- provide you with information which we believe may interest you;
- effectively communicate with you;
- respond to your requests and inquiries;
- provide you with products and services;
- provide your healthcare provider with information related to your use of the Services;
- provide you with updates and options about our products and services;
- analyze our customers' experiences with our products and services, including the Services described above; and
- improve our products and services.

Renovia may also use or disclose your Personal Data to third parties if we believe that such disclosure is:

- required by law;
- necessary to fulfill the above purposes;
- necessary to implement and/or enforce our Terms of Use agreement;
- necessary to protect the rights, property or personal safety of another Renovia user, Website visitor, other users of the Services, or any member of the public; or
- In order to operate the Services, fulfill the purposes set out above or otherwise deliver a product or service, we may sometimes share your Personal Data with an affiliate, agent, service provider, distributor, pharmacy, healthcare provider or other company in a business relationship with Renovia. When we use another company to perform a function of this nature, we endeavor to provide them only with the information that they need to perform their specific function and we instruct them to limit their use of the Personal Data accordingly.

As we develop our business, we might sell or buy businesses or assets. In the event of a corporate sale, merger, reorganization, dissolution or similar event, Personal Data may be part of the transferred assets. Except as described in this Privacy Policy or permitted by law, we will not otherwise disclose your Personal Data to other companies without your consent.

Additional Uses and Disclosures of Your Personal Information

We may also use or disclose your Personal Data in the following circumstances:

- **Required by Law**: We may use or disclose your Personal Data to the extent that applicable law requires the use or disclosure of such Personal Data. Where the use and/or disclosure of Personal Data is by law, the use or disclosure will be made in compliance with the law and will be limited to the relevant requirements of the law. You will be notified, as required by law, of any such uses or disclosures.
- **Public Health**: We may disclose your Personal Data for public health activities and purposes to a public health authority that is permitted by law to collect or receive the information. The disclosure will be made for the purpose of preventing or controlling disease, injury or disability. We may also disclose your Personal Data, if directed by the public health authority, to a foreign government agency that is collaborating with the public health authority.
- **Communicable Diseases**: We may disclose your Personal Data, if authorized by law, to a person who may have been exposed to a communicable disease or may otherwise be at risk of contracting or spreading the disease or condition.
- **Health Oversight**: We may disclose Personal Data to a health oversight agency for activities authorized by law, such as audits, investigations, and inspections. Oversight agencies seeking this information include government agencies that oversee the healthcare system, government benefit programs, other government regulatory programs and civil rights laws.
- **Food and Drug Administration**: We may disclose your Personal Data to a person or company as directed or required by the Food and Drug Administration: (i) to collect or report adverse events (or similar activities with respect to food or dietary supplements), product defects or problems (including problems with the use or labeling of a product), or biological product deviations, (ii) to track FDA-regulated products, (iii) to enable product recalls, repairs or replacement, or look back (including locating and notifying individuals who have received products that have been recalled, withdrawn, or are the subject of look back), or (iv) to conduct post-marketing surveillance.
- **Legal Proceedings**: We may disclose your Personal Data in the course of any judicial or administrative proceeding (i) in response to an order of a court or administrative tribunal (to the extent such disclosure is expressly authorized), and (ii) in certain conditions in response to a subpoena, discovery request or other lawful process, after we receive satisfactory assurance that the party seeking the information has reasonably attempted to notify you of the request or has reasonably attempted to secure a qualified protective order (in a court or administrative tribunal, or by stipulation) to limit disclosure of your Personal Data.
- **Law Enforcement**: We may disclose Personal Data, as long as applicable legal requirements are met, for law enforcement purposes. These law enforcement purposes include: (i) legal processes otherwise required by law, (ii) limited information requests for identification and location purposes, (iii) pertaining to victims of a crime, (iv) suspicion that death has occurred as a result of criminal conduct, (v) in the event that a crime occurs on the premises of the practice, and (vi) medical emergency in which it is likely that a crime has occurred.
- **Research**: We may disclose your Personal Data to researchers when their research has been approved by an institutional review board that has reviewed the research proposal and established protocols to ensure the privacy of your Personal Data.
- **Criminal Activity**: Consistent with applicable federal and state laws, we may disclose your Personal Data, if we believe the use or disclosure is necessary to prevent or lessen a serious and imminent threat to the health or safety of a person or the public. We may also disclose Personal Data if it is necessary for law enforcement authorities to identify or apprehend an individual.
- **Military Activity and National Security**: When the appropriate conditions apply, we may use or disclose Personal Data of individuals who are Armed Forces personnel (i) for activities deemed necessary by appropriate military command authorities; , or (ii) to foreign military authority if you are a member of that foreign military service. We may also disclose your Personal Data to authorized federal officials for conducting national security and intelligence activities.

- **Workers' Compensation**: We may disclose your Personal Data as authorized to comply with workers' compensation laws and other similar legally-established programs.
- **Inmates**: We may use or disclose your Personal Data to a correctional institution or law enforcement official if you are an inmate of a correctional facility and your physician created or received your Personal Data in the course of providing care to you, and disclosure is necessary for (i) providing you with health care; (ii) the health and safety of you, other inmates, or others at the correctional institution; or (iii) the administration and maintenance of the safety, security, and good order of the correctional institution.
- **Required Uses and Disclosures**: Under the law, we must make disclosures to you when required by the Secretary of the Department of Health and Human Services to investigate or determine our compliance with the requirements of the Health Insurance Portability and Accountability Act of 1996 and its implementing regulations ("**HIPAA**").
- **Non-identifiable Information**: We may use or disclose your Personal Data if we have removed from it any information that is personally identifiable to you.

When We Will Contact You

- We may contact you at the email address, mail address, telephone number, usernames or other contact information which you supply us in order to provide you with:
- Updated information about the Services;
- Updated information about Renovia;
- Updated options and information about our products and services;
- Other information that you have chosen to receive; and
- A response to your request(s).

What Are Your Choices?

You can use certain of the Services without providing any Personal Data. However, you may not be able to obtain certain products or services from Renovia including, without limitation, the *leva* and *your leva* mobile applications that are required in order to use the *leva* Pelvic Digital Health System and *leva* Pelvic Digital Therapeutic, respectively.

Storage and Security of Your Personal Data

At Renovia, we endeavor to take reasonable steps to keep secure any Personal Data that we hold about you, and to keep such Personal Data accurate and up to date. Access to your Personal Data is restricted within Renovia to employees, agents, affiliates or service providers, who have specific authorization. Since no data transmission over the Internet can be guaranteed to be totally secure, we do not promise that the information you provide to us will always be totally secure. Renovia disclaims any responsibility for events arising from unauthorized access to your Personal Data.

Third-Party Websites

Renovia may offer certain Services through third-party websites or provide links to third-party websites for your convenience. These third-party websites are not under our control, and we are not responsible for the conduct of or content provided by third-party websites. Before disclosing your Personal Data on any other website, we advise you to examine the terms and conditions and privacy policy of each of those websites. For more information concerning links to third-party websites, see the Terms of Use applicable to the Services. Links to third-party websites do not constitute or imply an endorsement by Renovia.

Exclusions

This Privacy Policy shall not apply to any unsolicited information you provide to Renovia through your use of the Services or through any other means. This includes, but is not limited to, information posted to any public areas of the Services, any ideas for new products or modifications to existing products, and other unsolicited submissions (collectively, "**Unsolicited Information**"). All such Unsolicited Information shall be deemed to be non-confidential and Renovia shall be free to reproduce, use, disclose, and distribute such Unsolicited Information to others without limitation or attribution.

Children's Online Privacy Protection Act

The Services are intended for adult use in the United States of America. No information should be submitted to or posted to any of the Services by users under 18 years of age without the consent of their parent or legal guardian. We encourage parents and legal guardians to monitor their children's Internet usage and to help enforce our Privacy Policy by instructing their children to never provide Personal Data on any of the Services without their permission. If you have reason to believe that a child under the age of 18 has provided Personal Data to Renovia through the use of any of the Services without the consent of their parent or legal guardian, you may contact us. If you have reason to believe that a child under the age of 13 has provided Personal Data to Renovia through the use of any of the Services without the consent of their parent or legal guardian, you may contact us and we will endeavor to delete that information from our records.

California "Do Not Track" Disclosure

In accordance with the California Online Privacy Protection Act ("**CalOPPA**") Amendment of 2013, we inform you that Renovia does not respond to Do Not Track requests or signals at this time.

What to Do If You Have a Problem or Question

If Renovia becomes aware of any ongoing concerns or problems with the Services, we will take these issues seriously and work to address these concerns or problems when possible. If you have any further questions relating to our Privacy Policy, or if you have a problem or complaint, please contact us by writing to:

Renovia Inc.
Attn: Privacy Officer
263 Summer Street
Boston, Massachusetts 02210
Contact@RenoviaInc.com
